# The NeuroEpiTool—A digital tool to ascertain neurological outcomes in population-based studies

**DOI:** 10.1016/j.isci.2026.117977

**Published:** 2026-10-25

**Authors:** Vasco Ribeiro Ferreira, Stefan Malinovski, Zara Belcheva, Dogukan Tuna, Wieke Bouwes, Paolo Ragonese, Richard Houeze, Francesca Madonia, Irene Assessou, Fadi Mohsen, Pierre-Marie Preux, Dieu Donné Gnonlonfoun, Constant Kodjo Adjien, Tim van Zutphen, Esther Metting, Valentina Gallo

**Affiliations:** 1 Department of Sustainable Health, University of Groningen Campus Fryslân, Leeuwarden, the Netherlands; 2 Bernoulli Institute, University of Groningen, Groningen, the Netherlands; 3 University Medical Center Groningen, Groningen, the Netherlands; 4 Department of Biomedicine, Neurosciences and Advanced Diagnostics, University of Palermo, Palermo, Italy; 5 Inserm U1094, IRD U270, University Limoges, EpiMaCT - Epidemiology of Chronic Diseases in Tropical Areas, Institute of Epidemiology and Tropical Neurology, Omega Health, Limoges, France; 6 Laboratory of Epidemiology of Chronic and Neurological Diseases, University of Abomey-Calavi, Cotonou, Benin; 7 Department of Neurology, CNHU-HKM of Cotonou, Cotonou, Benin; 8 Faculty of Economics and Business, University of Groningen, Groningen, the Netherlands

**Keywords:** neuroepidemiology, neurological disorders, digital health, eHealth

## Abstract

Collecting data on signs and symptoms of neurological disorders requires extensive resources, limiting feasibility. This study reports on the NeuroEpiTool, a digital tool designed to facilitate data collection in neuroepidemiological studies. Eighty participants (47.5% female, *n* = 38) with and without neurological disorders were evaluated using the NeuroEpiTool. Inter-rater reliability was assessed between two health professionals using the tool. Additionally, convergent validity and usability were explored. Moderate to strong inter-rater agreement was observed across most neurological tests. The 30-s chair stand test, though not typically part of a standard neurological exam, showed one of the highest agreement levels (κ = 0.82, *p* < 0.001). Feedback from physicians suggested that the NeuroEpiTool closely resembled a standard neurological examination. Usability had an average of 81.88 ± 10.87. Inter-rater reliability scores support the potential for data collection by non-experts. Future studies should pursue large-scale validation and real-world implementation to support electronic health (eHealth) solutions in neuroepidemiology.

## Highlights

• The NeuroEpiTool guides data collection for non-neurologist health professionals
• The tool was tested in two locations, supporting its applicability in diverse populations
• Moderate to strong inter-rater agreement was observed between assessors for most tests
• The NeuroEpiTool supports large-scale neuroepidemiological research in low-resource settings

## Graphical abstract

**Figure:**
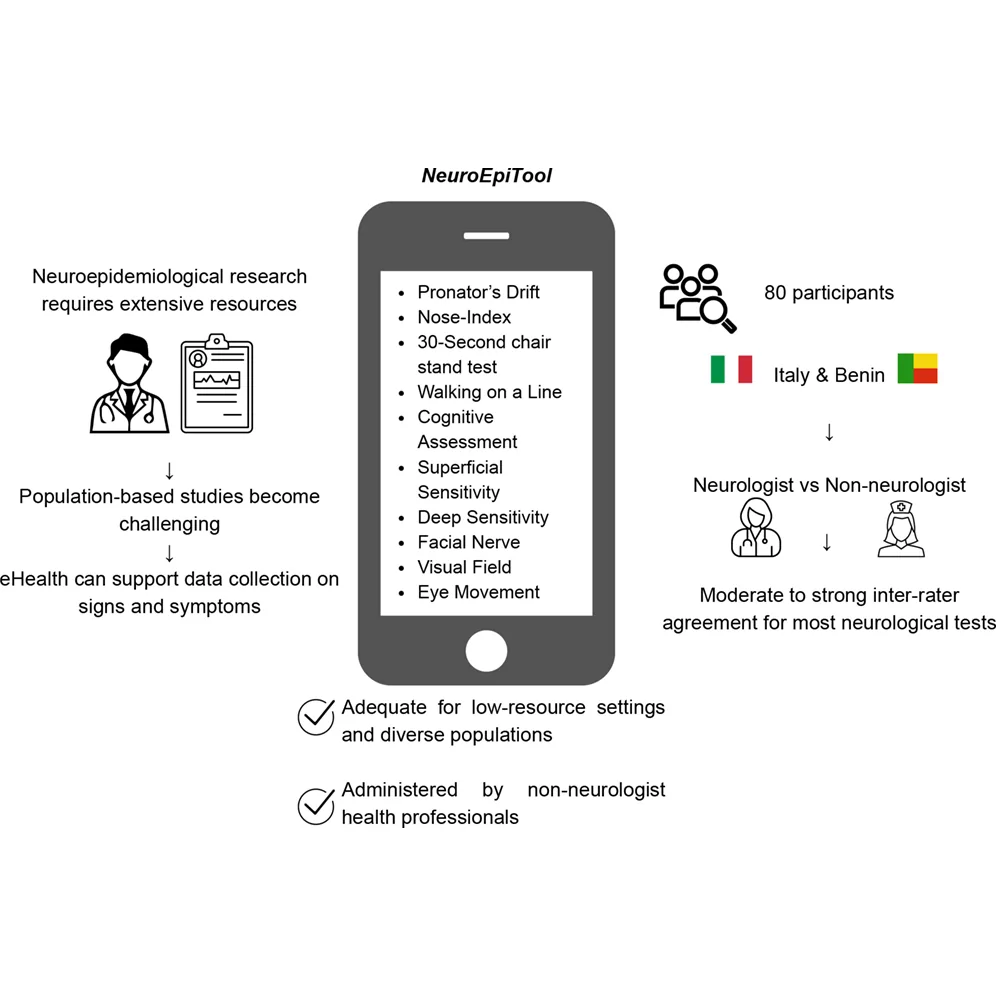

## Teaser

Health sciences; Medicine; Medical specialty; Internal medicine; Neurology; Public health

## Introduction

Neurological disorders, their prevalence, and their contribution to the global disease burden have become increasingly evident, with an estimated 3.4 billion (3.20–3.62) cases worldwide.^1^ Efforts from both clinical and research perspectives have been aimed at mitigating this burden by unravelling the complexity of neurological disorders and their risk profiles; however, the role of several exposures (e.g., pesticides, herbicides, and solvents) remains unclear, as seen in the case of glyphosate and Parkinson’s disease.^2^^,^^3^^,^^4^ At the individual level, early detection is critical for minimising the impact on neurological function.^5^ Similarly, at the population level, comprehensive cohort studies exploring causal relationships are essential for developing effective preventive strategies.^6^^,^^7^^,^^8^^,^^9^ However, studying neurological disorders and their elusive risk profiles carries significant challenges, particularly in securing the necessary resources for performing neuroepidemiological research outside clinical environments and in low-resource settings.^10^

Electronic health (eHealth) has shown promising potential in healthcare and health research, benefiting health professionals, patients, and caregivers.^11^^,^^12^ However, eHealth tools for research on signs and symptoms of neurological disorders are relatively limited: the tools typically target specific disorders or a narrow neurological domain (e.g., only motor, cognitive, or coordination impairments). Reviews of eHealth software and hardware that assess neurological function stressed the need for a unified tool to capture a wide range of neurological signs and symptoms.^13^^,^^14^ Furthermore, eHealth tools designed explicitly for research purposes remain scarce compared to those developed for clinical applications. Currently, eHealth mainly focuses on clinical applications at the individual level (e.g., diagnosing, guiding therapy, classifying, and monitoring symptoms), often overlooking its potential advantages for research contexts, such as collecting data at a large scale.^13^ While numerous eHealth tools are being constantly developed, only a fraction are successfully implemented, effective, and sustainable.^15^

Implementation challenges have remained a central issue in the eHealth field in recent years, particularly in low-resource settings, where coordinating with local institutions, researchers, and other professionals (e.g., health workers and informatics experts) can be challenging.^16^^,^^17^ Proposed solutions to overcome implementation challenges include frameworks that suggest considering key factors influencing development, implementation, and evaluation from the initial stages of tool development. For example, the CeHRes roadmap emphasises the importance of engaging multiple stakeholders from the outset and recommends continuous feedback, usability testing, and structured implementation strategies, with a focus on real-world adoption and iterative improvements.^18^^,^^19^

In response to the identified challenges and gaps, we developed a new eHealth tool, the NeuroEpiTool, aligned with the CeHRes roadmap. We engaged relevant stakeholders early in the development process and prioritised maintenance strategies to maximise the long-term sustainability of the new tool. The NeuroEpiTool adapts a digital neurological examination to facilitate data collection by non-neurologists. The tool focuses on sensitivity, capturing signs and symptoms across a wide range of neurological disorders. The aim is not to diagnose or classify at an individual level but rather to explore patterns in a population. Unlike most existing tools, the NeuroEpiTool is designed explicitly for neuroepidemiological research, allowing for data collection without the need for an expert to be present.

This paper details the structure and internal validation study of the first version of the NeuroEpiTool. One of the primary objectives of the internal validation was to evaluate the agreement between neurologists and non-neurologist health professionals using the NeuroEpiTool. Thus, we expect that most neurological tests in the NeuroEpiTool demonstrate moderate to substantial agreement between the assessors. Similarly, we anticipate that users of the NeuroEpiTool will report high usability, following the CeHRes roadmap to increase uptake.

## Results

### Inter-rater reliability

Participant demographic data is provided in Table 1. A flowchart detailing the sections of the NeuroEpiTool is displayed in Figure 1. According to the analysis, three tests in the NeuroEpiTool demonstrated strong agreement between the two assessors: the 30-s chair stand (κ = 0.82, *p* < 0.001), the superficial sensitivity (κ = 0.83, *p* < 0.001), and the visual field (κ = 0.80, *p* < 0.001). Three other tests demonstrated moderate agreement: the walking on a line (κ = 0.69, *p* < 0.001), the cognitive assessment (κ = 0.74, *p* < 0.001), and the facial nerve (κ = 0.60, *p* < 0.001). The pronator’s drift of the upper limbs, the nose-index, and the eye movement tests revealed weak agreement. The complementary tests (i.e., the pronator’s drift of the lower limbs and the deep sensitivity of the upper and lower limbs) showed no significance, possibly due to insufficient data. A detailed view of the values for each test is provided in Table 2, and a visual overview of the Cohen’s kappa for the main tests is available in Figure 2.

**Table 1:** Participant demographics by data collection site

Characteristic	Italy (*n* = 29)	Benin (*n* = 51)	Overall (*n* = 80)
Age, years, mean (SD)^a^	54.2 (25.5)	53.3 (14.8)	53.6 (19.2)
Female sex, *n* (%)	21 (72.4)	17 (33.3)	38 (47.5)
Education, years, mean (SD)	13.2 (4.6)	13.0 (5.7)	13.1 (5.3)
Diagnosis, *n* (%)			
None (non-impaired)	3 (10.3)	10 (19.6)	13 (16.3)
Multiple sclerosis	24 (82.7)	–	24 (30.0)
Stroke	–	18 (35.3)	18 (22.5)
Epilepsy	–	5 (9.8)	5 (6.3)
Parkinson’s disease	–	3 (5.9)	3 (3.8)
Other^b^	2 (6.9)	13 (25.5)	15 (18.8)

a Age was estimated from year of birth and year of assessment because exact birth dates were not collected.

b Other diagnoses include migraines, vestibular disorders, essential tremor, and dementia.

**Figure 1:**
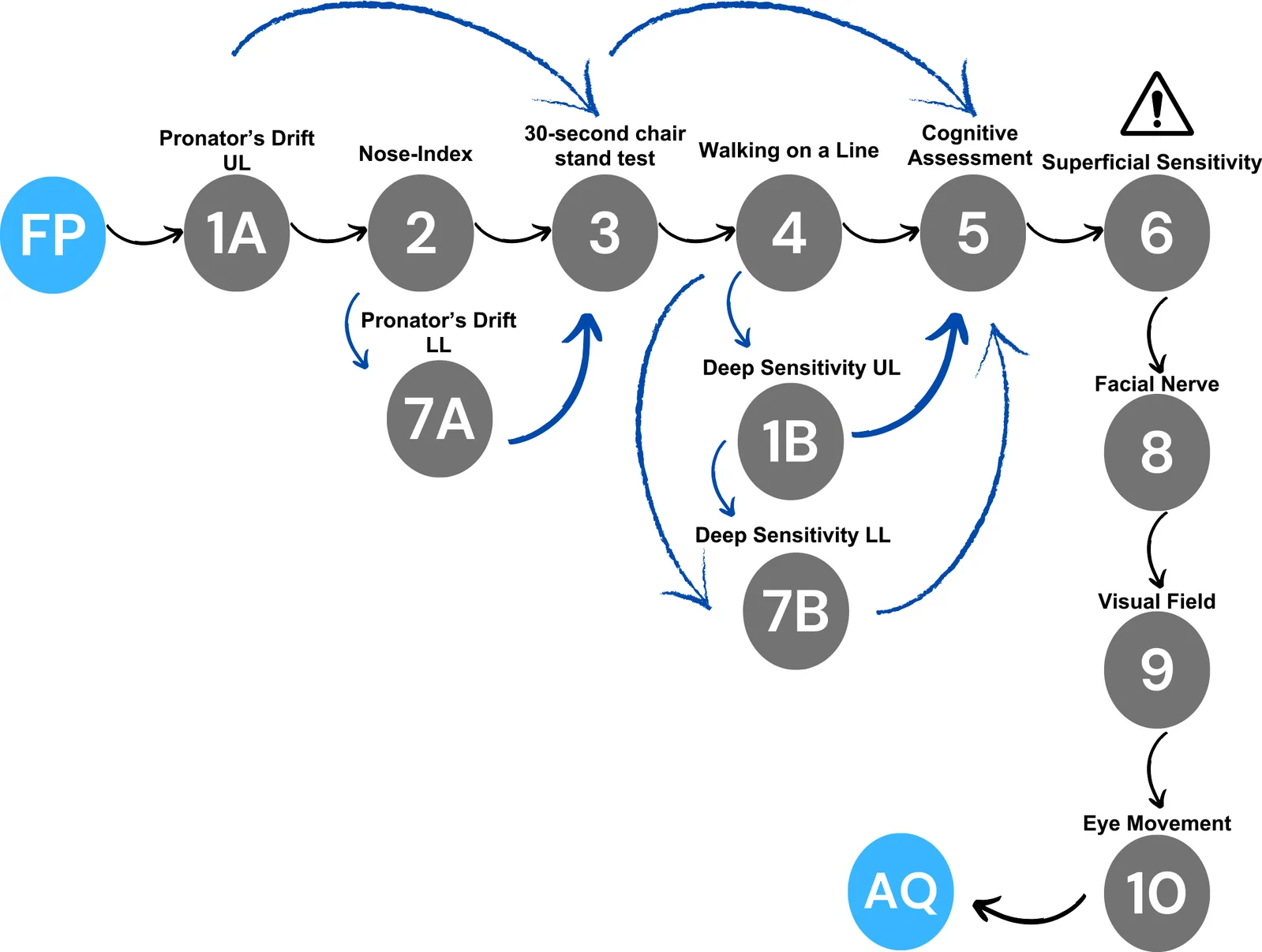
Step-by-step flowchart main overview for the sections in the NeuroEpiTool and how they connect to one another Each numbered section corresponds to a neurological test. Section 6 warns the assessor to ensure the participant is okay with being touched. Section 10 corresponds to the last test and leads into the questions section. FP, front page; UL, upper limb; LL, lower limb; AQ, anamnestic questions.

**Table 2:** Level of agreement statistics obtained with Cohen’s kappa

Neurological test	Cohen’s kappa (κ)	CI	*p* value	*n*
**Main tests**				

Pronator’s drift Upper limb	0.57	[0.38, 0.75]	0.001	80
Nose-index	0.51	[0.17, 0.85]	0.001	54
30-s chair stand test	0.82	[0.69, 0.96]	0.001	80
Walking on a line	0.69	[0.52, 0.86]	0.001	71
Cognitive assessment	0.74	[0.56, 0.92]	0.001	80
Superficial sensitivity	0.83	[0.69, 0.97]	0.001	80
Facial nerve	0.60	[0.40, 0.80]	0.001	80
Visual field	0.80	[0.59, 1.02]	0.001	80
Eye movement	0.54	[0.27, 0.82]	0.001	80

**Complementary tests**				

Pronator’s drift Lower limb	0.26	[−0.18, 0.69]	0.26	19
Deep sensitivity Upper limb	–	–	–	1
Deep sensitivity Lower limb	0.39	[0.01, 0.76]	0.06	23

Additional complementary tests were only performed on a few selected participants who demonstrated impairment on previous tests. If κ ˂ 0.20, it indicates no agreement; if 0.21 ≤ κ ≤ 0.39, there is minimal agreement; if 0.40 ≤ κ ≤ 0.59, there is weak agreement; if 0.60 ≤ κ ≤ 0.79, there is moderate agreement; and if κ ≥ 0.80, there is strong agreement.

**Figure 2:**
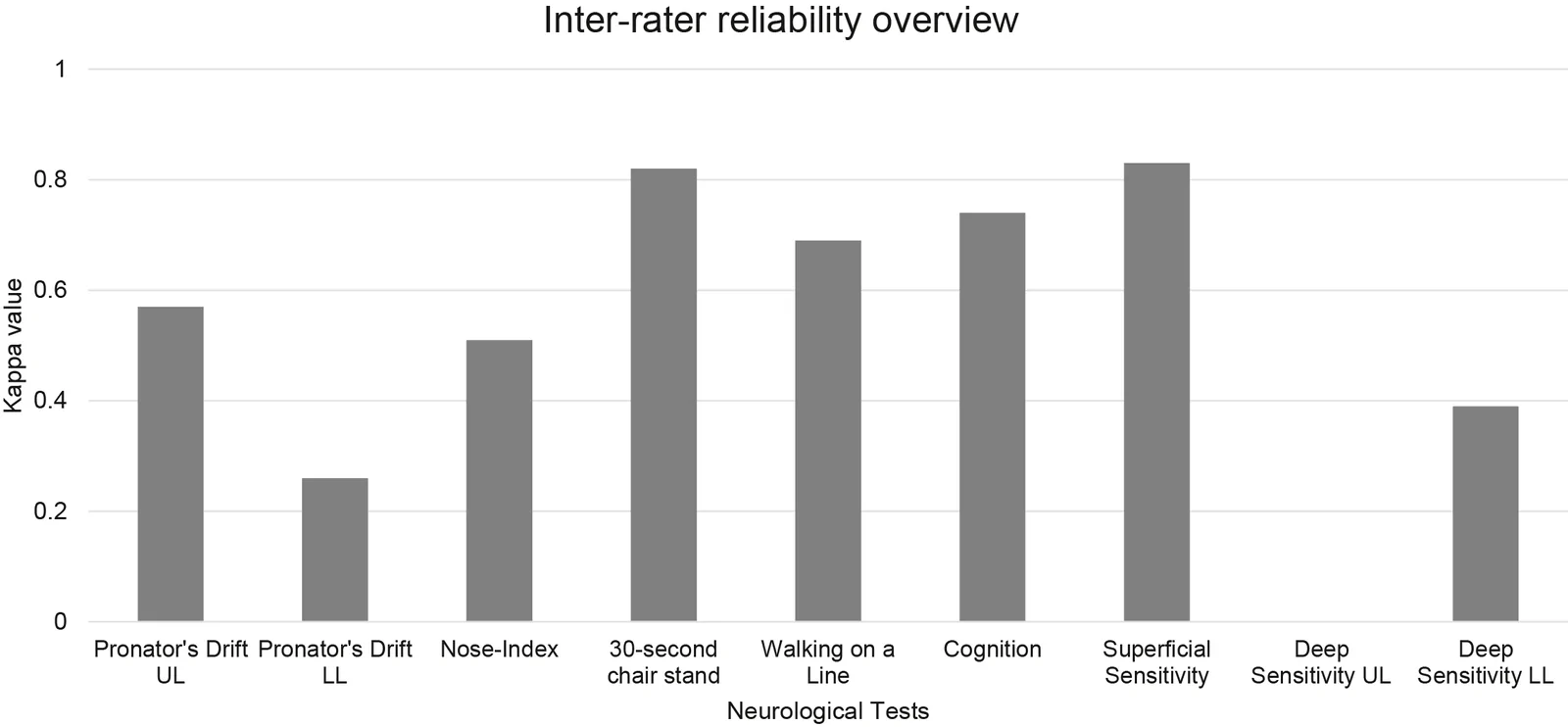
Level of agreement (inter-rater reliability) across the main neurological tests, obtained with Cohen’s kappa A larger value in a test indicates stronger agreement between the two assessors who used the NeuroEpiTool. UL, upper limb; LL, lower limb.

### Convergent validity and usability

The two neurologists who collected data with the NeuroEpiTool provided promising feedback regarding its completeness, limitations, and overall appreciation. Table 3 provides the complete responses from the neurologists, by question.

**Table 3:** Neurologists' responses to questions evaluating the NeuroEpiTool’s clinical resemblance and assessment accuracy

Question	Neurologist 1 (Italy)	Neurologist 2 (Benin)
To what extent was the NeuroEpiTool able to capture the actual neurological status of the participants you assessed? In other words, does it provide, on average, a fair picture of the neurological status?	yes, but limitations are possible for people with very slight symptoms	yes
Do you perceive the NeuroEpiTool as being able to capture the impairment of some specific neurological conditions better than others? If yes, which?	visual impairment could represent a weakness	some neurological conditions are well captured (palsies, headache, myopathy, balance disorders, cerebellar syndrome, and cognitive disorders)
Do you have any additional comments or feedback on the NeuroEpiTool?	the tool should be able to interact with other tools	the NeuroEpiTool is a very good tool for non-neurological health professionals
The NeuroEpiTool resembled a standard neurological examination (Likert-type scale)	somewhat agree	somewhat agree

Regarding usability, the average score in the System Usability Scale (SUS) was 81.88 ± 10.87, indicating an overall high usability score for the NeuroEpiTool. Individual scoring is provided in Table 4.

**Table 4:** Usability scores (SUS) for the NeuroEpiTool with varying roles and geographical locations

Assessor	Neurologist 1 (Italy)	Neurologist 2 (Benin)	Psychologist (Italy)	Nurse (Benin)
SUS score	97.5	77.5	80.0	72.5

## Discussion

This study reported the development and internal validation of the NeuroEpiTool, a digital research tool designed to collect signs and symptoms of neurological disorders without the need for a neurologist. Data collection was conducted in both the Global North and South, with the primary aim of assessing inter-rater reliability between a neurologist and a non-neurologist health professional using the tool. Convergent validity and usability were also explored. Overall, the findings support the reliability, usability, and construct validity of the NeuroEpiTool.

### Inter-rater reliability

Most of the main neurological tests demonstrated moderate to strong agreement. Three complementary tests (the pronator’s drift of the lower limbs and the deep sensitivity of the upper and lower limbs) appeared to demonstrate minimal agreement, likely due to the limited data available. Interestingly, one of the tests with the highest level of agreement, the 30-s chair stand, was not usually part of the routine neurological examination, as stated by the neurologists. However, as the data indicate, it appears to be a highly reliable test that non-neurologists can perform and interpret. Some inconsistencies were found between the assessors’ interpretations. For instance, in the pronator’s drift of the upper limbs, the neurologist noted one arm falling, while the non-neurologist observed pronation, both recognising impairment but describing it differently. The differences in interpretation may stem from symptom fluctuation, as some neurological disorders can manifest differently throughout the same day, particularly motor symptoms and fatigue.^20^^,^^21^ In other tests, such as the eye movement and nose-index tests, the level of agreement was lower, potentially due to overdiagnosis by non-neurologists, who may have flagged minor asymmetries as impairments. Thus, a non-neurologist should interpret these two tests cautiously, as their interpretation requires significant expertise and formal training. Nonetheless, the inclusion of these tests in the NeuroEpiTool aligns with the aim of a comprehensive screening assessment, thereby avoiding the risk of missing impairments.

Few previous studies have compared a neurologist’s assessment with that of other health professionals. Studies focus mainly on validated clinical measures (e.g., the Unified Parkinson’s Disease Rating Scale [UPDRS]) rather than digital tools. A paper on the UPDRS motor exam described moderate to substantial agreement between the assessment of neurologists and nurses.^22^ Another paper on stroke assessment with the Oxfordshire Community Stroke Project (OCSP) revealed fair to moderate agreement between nurses and neurologists.^23^ A study comparing neurologists with physiotherapists highlighted low inter-rater agreement for the pronator’s drift, nose-index, and pupil symmetry tests.^24^ Eye-related assessments, such as pupil symmetry and eye movement, appear particularly challenging for non-neurologists. Conversely, the tandem gait test has been shown to demonstrate a high level of agreement in the literature, which aligns with our findings for the walking on a line test in the NeuroEpiTool.^24^ In addition, tests assessing observable signs such as gait, balance, and coordination show better consistency when compared to elicitable signs (e.g., strength, tone, and reflexes), supporting our test selection.^25^ These previous studies and our own findings support the premise that, with appropriate training, non-neurologist health professionals can fairly rate and interpret signs and symptoms of neurological disorders.

### Convergent validity and usability

Neurologists generally agreed that the NeuroEpiTool resembled a standard neurological examination. They noted, however, that subtle signs might go undetected by the NeuroEpiTool, particularly in conditions like multiple sclerosis or epilepsy. According to the neurologists, there is high relevance in including anamnestic questions: even though the participant may not present with any signs or symptoms at the time of these questions, they complement the neurological examination and add relevant information that might otherwise not be captured by the tests. Visual impairment was also noted as a potential confounder, but the tool accounts for uncorrected visual and hearing impairments on its front page. Overall, the feedback from the neurologists was very positive and encouraging, suggesting that the NeuroEpiTool should be continued, improved upon, and deployed widely.

Usability scores were moderate to high for all assessors. There was a slight increase in usability for neurologists, possibly due to their greater familiarity with the instructions, tests, and rating systems. Non-neurologists may have been less confident administering the tests, which was reflected in lower usability scores. Assessors in Italy scored higher on usability, which may be attributed to higher digital literacy levels within the Global North compared to the Global South^26^; however, the French translation may have also influenced usability. A previous study found that medical professionals perceived the usability of medical record systems differently from nurses, with these differences varying across hospital and primary care sectors, as well as between countries, possibly due to differing practices and systems in each country.^27^ The overall usability scores were better than expected, indicating that the NeuroEpiTool does not require an exceptionally high level of digital literacy to be administered effectively.

### NeuroEpiTool for neuroepidemiological studies

The NeuroEpiTool reported in this paper has an innovative character, as it can capture a wide range of signs and symptoms across several neurological disorders, particularly those that affect gait, balance, cognition, and facial nerves. It is reliable for administration and interpretation by non-neurologists, requiring only a smartphone, pen, and internet connection, making it suitable for low-resource settings. In contrast to many existing digital tools designed to support the diagnosis, classification, or monitoring of specific neurological disorders, the NeuroEpiTool provides a digital assessment of neurological function that can be administered by non-neurologist healthcare professionals for research. While very mild symptoms may be misinterpreted in specific tests, the tool meets its goal of facilitating data collection on signs and symptoms. Future studies should focus on validating the NeuroEpiTool in large-scale epidemiological studies (i.e., cohort studies), formally assessing sensitivity against a standard neurological examination, and exploring its use by other trained personnel, such as epidemiologists, to support exposure-outcome studies.

### Limitations of the study

The overall positive results hold promising expectations for the future of the NeuroEpiTool and digital health, as well as its implementation in real-world settings and beyond clinical environments. One major limitation of this study is that the assessors were often not blinded to the participants’ diagnoses; since data collection was primarily conducted during neurologist consultations, the neurologist was already aware of the participants’ neurological status. We also did not assess prior experience with eHealth or the level of digital literacy of the assessors, which may have influenced the usability scores. In addition, a relatively small sample affected the data in some of the neurological tests, and the fact that the NeuroEpiTool was translated into French and used in two different languages might have hindered entirely fair comparisons. While the lack of enough data limited comparability across the whole test set, it reflects real-world clinical reasoning, where additional tests are only conducted when initial performance indicates potential impairment. Given that the comparison was between different types of assessors, the overall findings support a good level of agreement between experts and non-experts in interpreting signs and symptoms of neurological disorders with the NeuroEpiTool.

Misclassification is possible for items demonstrating lower inter-rater agreement, likely reflecting the greater complexity of some neurological tests, differences in interpretation, and the limited training of non-specialist assessors. However, because the NeuroEpiTool is intended as a research rather than a clinical tool, identifying potential impairment is prioritised over guiding a diagnosis. Consequently, occasional false-positive findings are preferable to missing potential neurological impairment. Individuals identified as having possible impairment should subsequently undergo a standard neurological examination by a trained neurologist.

### Conclusion

This study reported on the development and internal validation of a new digital research tool to capture signs and symptoms of neurological disorders without the need for a neurologist. Moderate to excellent agreement was observed across most of the main neurological tests. Similarly, high usability was reported among all assessors using the NeuroEpiTool. Neurologists also perceived the NeuroEpiTool to resemble a standard neurological examination, albeit with some expected limitations. The findings of this study highlight the potential of digital health tools to support data collection and assessments in neuroepidemiological research.

## Resource availability

### Lead contact

Further information and requests for resources should be directed to and will be fulfilled by the lead contact, Vasco Ribeiro Ferreira (vasco.ferreira95@gmail.com).

### Materials availability

This study did not generate new unique reagents.

### Data and code availability

• Data reported in this paper can be shared by the lead contact upon request.
• Accession codes to the materials used can be found in the key resources table. The source code is publicly available on GitHub (https://github.com/laexdt/eHealth-research-tool).
• The NeuroEpiTool is freely accessible at https://neuroepitool.web.rug.nl/. Any additional information required to reanalyze the data reported in this paper is available from the lead contact upon request.

## Acknowledgments

This article is part of a larger funded PhD project at the University of Groningen. Additional funding was acquired from the Young Academy Groningen (YAG) seed grants. The authors would like to thank the EpiMaCT for their crucial support in the project, as well as all the participants and health staff who contributed to the study’s success.

## Author contributions

V.R.F. was responsible for conceptualization, methodology, validation, formal analysis, investigation, data curation, visualization, funding acquisition, and writing the original draft, review, and editing. S.M., Z.B., and D.T. were responsible for the software and reviewing. W.B. contributed to the writing of the original draft, review, and editing. P.R. and R.H. provided supervision and resources, contributed to the investigation, and reviewed the manuscript. F. Madonia and I.A. contributed to the investigation, data curation, and reviewing of the manuscript. F. Mohsen was responsible for software, supervision, and reviewing the manuscript. P.-M.P., D.D.G., and C.K.A. contributed resources, provided project administration, and reviewed the manuscript. T.v.Z., E.M., and V.G. were the main supervisors and project administrators; they also contributed to the review and editing of the manuscript.

## Declaration of interests

The authors declare no competing interests.

## STAR★Methods

### Key resources table

**Table:** 

REAGENT or RESOURCE	SOURCE	IDENTIFIER
**Deposited data**		

NeuroEpiTool source code	GitHub	https://github.com/laexdt/eHealth-research-tool

**Software and algorithms**		

NeuroEpiTool	This study	https://neuroepitool.web.rug.nl/
Qualtrics	Qualtrics	RRID:SCR_016728
IBM SPSS Statistics v28	IBM	RRID:SCR_016479

**Other**		

Neurological Examination Made Easy	Fuller	ISBN 13: 9780702051777

### Experimental model and study participant details

Ethical approval was obtained from the leading research centers’ ethical committees (i.e., the University of Groningen Campus Fryslân – approval number cf-ucg.2024.003 – and the University Medical Center Groningen), as well as from each data collection research center.

Eighty participants with a balanced sex distribution (47.5% female, *n* = 38) living with and without neurological disorders (83.8% impaired, *n* = 67) were evaluated with the NeuroEpiTool twice, once with each assessor. Assessors were blinded to each other’s assessments, but not to the participant’s diagnosis. Non-impaired participants were mainly recruited from patient companions or staff. Data were collected from two health centers: the Policlinico Universitario Paolo Giaccone in Italy (*n* = 29) and the National University Hospital Center Hubert Koutoukou MAGA (CNHU-HKM) in Benin (*n* = 51). We chose these two different centers to capture a broader range of participants from diverse geographical, cultural, and social contexts, and to facilitate a better understanding of how the NeuroEpiTool performs in different settings. Consent was given via a written informed consent form. Participant characteristics are displayed in Table 1.

### Method details

#### Context

The digital tool described in this paper, the NeuroEpiTool, originates from a larger project aimed at developing a tool for assessing neurological impairment at the population level, specifically designed for use by non-neurologists in neuroepidemiological research. In earlier phases of the project, a protocol and two systematic reviews were published to map existing eHealth software and hardware tools for evaluating one or more neurological functions.^13^^,^^14^^,^^28^ The systematic reviews provided an overview of barriers and facilitators to eHealth development and implementation for assessing neurological function, which were taken into account when developing our tool. A Delphi consensus study was conducted afterwards to determine the most appropriate screening tests and questions for capturing signs and symptoms of neurological disorders.^29^ Since non-neurologists will be the primary users of the tool, the neurological examination was deliberately organised by function rather than anatomical location to enhance clarity for non-experts (e.g., coordination impaired instead of cerebellum lesion). Furthermore, a panel of experts in neurology, epidemiology, and participant experience contributed to the Delphi study, ensuring a multidisciplinary perspective to refine the tool’s design. The NeuroEpiTool adapts a standard neurological examination into a semi-automated digital format, structured as a step-by-step process to be navigated by a trained assessor. The tool was developed following the principle of parsimony: if an item yields a positive result (i.e., flagged as impaired), complementary tests may be required to refine the identification of neurological impairment.

This paper describes the internal validation of the NeuroEpiTool prototype in Italy and Benin, two countries located in the Global North and the Global South, respectively. The internal validation primarily aimed to evaluate the accuracy of a non-expert’s performance compared to that of an expert, determining whether a non-neurologist could administer the tool and consistently interpret participants’ responses with the same accuracy as a neurologist. By surveying the assessors, we also investigated the usability and construct validity of the NeuroEpiTool and its resemblance to a standard neurological examination.

#### Selecting the content for the tool

The items identified in the Delphi study guided the design of several chart sections, each focused on exploring impairment and corresponding to a neurological examination test that reached consensus in the Delphi study.^29^ Experts in the neurological examination proposed neurological tests and questions, and experts in data collection and participant experience rated them based on their feasibility and interpretability for non-neurologists, as well as their acceptability to participants. The full criteria and details can be viewed in the published paper.^29^ The final selection from the Delphi study included ten neurological tests and seven anamnestic questions covering a broad range of signs and symptoms.

The neurological tests in the NeuroEpiTool include the Pronator’s Drift, the Nose-Index, the 30-s Chair Stand, Walking on a Line, a Cognitive Assessment, Superficial Sensitivity, Deep Sensitivity, Facial Nerve, Visual Field, and Eye Movement. Anamnestic questions complemented the assessments not covered by the tests. An additional anamnestic question assessing movement slowness was incorporated during subsequent expert feedback loops, after the Delphi study. Similarly, five additional anamnestic questions related to epilepsy were included based on a validated epilepsy questionnaire.^30^ The additional anamnestic questions were suggested by experts, as an attempt to capture any remaining signs and symptoms of neurological disorders that could potentially still be explored.

#### The NeuroEpiTool

The prototype of the NeuroEpiTool was designed using Qualtrics software, and later coded into the first version, which is hosted on a website (https://neuroepitool.web.rug.nl/). It is open source, with the possibility to access its source code, integrate it, or expand it. The NeuroEpiTool was built in a web-based format for easy accessibility and compatibility with most smartphones and tablets. Initially developed in English, it was later translated into French by a certified translator to meet the needs of users in Benin. To minimise privacy risks, the NeuroEpiTool does not collect directly identifiable personal information. Data collected is limited to sex, year of birth, years of education, and known diagnosis, where applicable. Following each assessment, data must be exported as a.csv file or otherwise permanently deleted. The exported data were stored on a secure server at the University of Groningen and handled in accordance with the General Data Protection Regulation (GDPR). As the NeuroEpiTool may be used in different jurisdictions, users are responsible for ensuring that data collection, storage, and processing comply with applicable local regulations and institutional requirements.

Since non-neurologists are the primary users of the tool, text instructions appear before the neurological tests to guide the assessor in conducting the assessment adequately. At the same time, they prevented the trained neurologists from deviating from the digital neurological examination in the NeuroEpiTool. The assessor is then guided through questions that help interpret the participant’s performance. The step-by-step flowchart was designed to administer specific tests only if preceding ones indicated a potential impairment, and a flowchart was built based on this parsimonious approach. Additionally, tests evaluating both upper and lower limb function were placed early in the sequence, ensuring that if a participant had a missing or immobile limb, subsequent tests would account for this limitation (e.g., if a participant cannot repeatedly sit and stand from a chair even with assistance, the walking in a line test would be deemed infeasible). The neurological tests were divided into main and complementary tests: complementary tests are only performed if specific main tests are impaired (Figure 1). The NeuroEpiTool was designed to maximise sensitivity and not specificity, meaning that it should correctly capture all individuals with impairment.

The flowchart followed an iterative process involving feedback loops between key stakeholders, including neuroscientists, epidemiologists, neurologists, and information technology (IT) specialists. While incorporating the designed elements into the tool, the IT team provided feedback on the flowchart, user input, potential implementation strategies, and alternative solutions when necessary. The neurological examination experts who participated in the Delphi study reviewed the semi-final flowchart, providing feedback on decision nodes, potential outcomes, and the overall rationale. The neurological test assessments in the NeuroEpiTool result in outcomes for each participant. These outcomes are based on a standard neurological examination, with the main outcome referring to impaired or non-impaired performance for each test. Additional outcomes include the allocation of impairment to at least one side of the body with three levels (i.e., left, right, or both); the categorisation of intensity according to the severity of impairment with three levels (i.e., mild, moderate, severe); and the attribution of a cause of impairment to one of the neurological functions (e.g., strength impairment of the upper limbs, coordination impairment in the lower limbs). The flowchart was built with several sections, each corresponding to a specific neurological test, and an additional section referring to the anamnestic questions.

To maximise tool homogeneity and usability, the input from the assessor to most questions is a choice between “yes” and “no”. This response format across sections helps ensure uniformity across assessors and promotes consistency. However, some sections may require additional input when performance is impaired (e.g., in Section Three – 30-s chair stand, the assessor specifies whether the participant can only perform the test with help, or without help but with difficulty, or is unable to perform it even with help). Although most input is given through single selection, there are a few instances in which multiple selection was implemented, where the assessor selects all options that apply to the participant’s performance (e.g., in section four – walking on a line, the participant may be unable to walk on heels while simultaneously unable to walk on their tiptoes).

Non-neurologist assessors were required to be healthcare professionals. Their training on the NeuroEpiTool was supported by a series of illustrated images and demonstration videos showing the correct performance of each neurological test, alongside examples of impaired performance. The *Neurological Examination Made Easy* by Fuller served as the reference for performance and interpretation. The training session lasted approximately 15 min, and a neurologist was present throughout the training to answer questions and provide clarification. The same training materials and procedures were used across both study sites to ensure consistency.

### Quantification and statistical analysis

#### Inter-rater reliability

We measured inter-rater reliability by comparing the assessments of a neurologist using the tool with those of a non-neurologist who also used the same tool. One neurologist collected data in each center, with one psychologist from the Italian center and one nurse from the Beninese center as non-expert assessors. The study was explained to participants by handing them an information sheet in their native language, and the neurologist answered any remaining questions. Each NeuroEpiTool assessment lasted approximately 15 min, totalling 30 min for each participant. All participant data were merged into a CSV file and handled in Excel.

#### Convergent validity and usability

By the end of data collection from each center, the neurologists answered three open-ended questions regarding their perceptions of the NeuroEpiTool. Both neurologists and non-neurologists completed a 5-min questionnaire on usability, the System Usability Scale (SUS), which consisted of 10 questions exploring the usability of a technological system through Likert-type scale answers ranging from 1 (strongly disagree) to 5 (strongly agree). The total score has a maximum of 100, with a cut-off of approximately 68 indicating reasonable usability and around 80 translating to high usability.^31^ An additional question was added to the neurologists’ questionnaire, inquiring about the resemblance of the NeuroEpiTool to a standard neurological examination. However, this question was merely qualitative, and no formal quantitative comparisons were made with a standard neurological examination.

#### Data analysis

The primary objective of the analysis was to assess the level of agreement between the two assessors who used the NeuroEpiTool: the neurologist and the non-neurologist. Cohen’s Kappa measure of agreement was calculated, a coefficient that takes into account the level of agreement between two assessors, while also accounting for the possibility of agreement by chance. The IBM SPSS Statistics software, version 28, was used for all data analysis. The reference values for Cohen’s Kappa in health studies indicate that a value below 0.20 signifies no agreement, while a value above 0.80 indicates strong agreement.^32^ Cohen’s Kappa was computed for each neurological test, based on the assessor’s interpretation of whether the participant’s performance was impaired or non-impaired (i.e., a binary outcome for each test). In total, 10 tests were part of the NeuroEpiTool examination, with two of them divided into Upper and Lower Limbs (i.e., the Pronator’s Drift and the Deep Sensitivity). Thus, 12 outcomes were computed for each participant. However, since three neurological tests were only performed conditionally, these tests lacked sufficient data to calculate Cohen’s Kappa appropriately.
