# Supplementary material for: The NeuroEpiTool—A digital tool to ascertain neurological outcomes in population-based studies
Source: iScience. 2026 Oct 25;29(11):117977. doi: 10.1016/j.isci.2026.117977 (PMC13722562; doi:10.1016/j.isci.2026.117977)
Supplement: Supplementary file 1 [file mmc1.pdf]

iScience, Volume *29*

Supplemental information

The NeuroEpiTool—A digital tool to ascertain neurological outcomes in population-based studies

Vasco Ribeiro Ferreira, Stefan Malinovski, Zara Belcheva, Dogukan Tuna, Wieke Bouwes, Paolo Ragonese, Richard Houeze, Francesca Madonia, Irene Assessou, Fadi Mohsen, Pierre-Marie Preux, Dieu Donné Gnonlonfoun, Constant Kodjo Adjien, Tim van Zutphen, Esther Metting, and Valentina Gallo

SUPPLEMENTAL MATERIAL

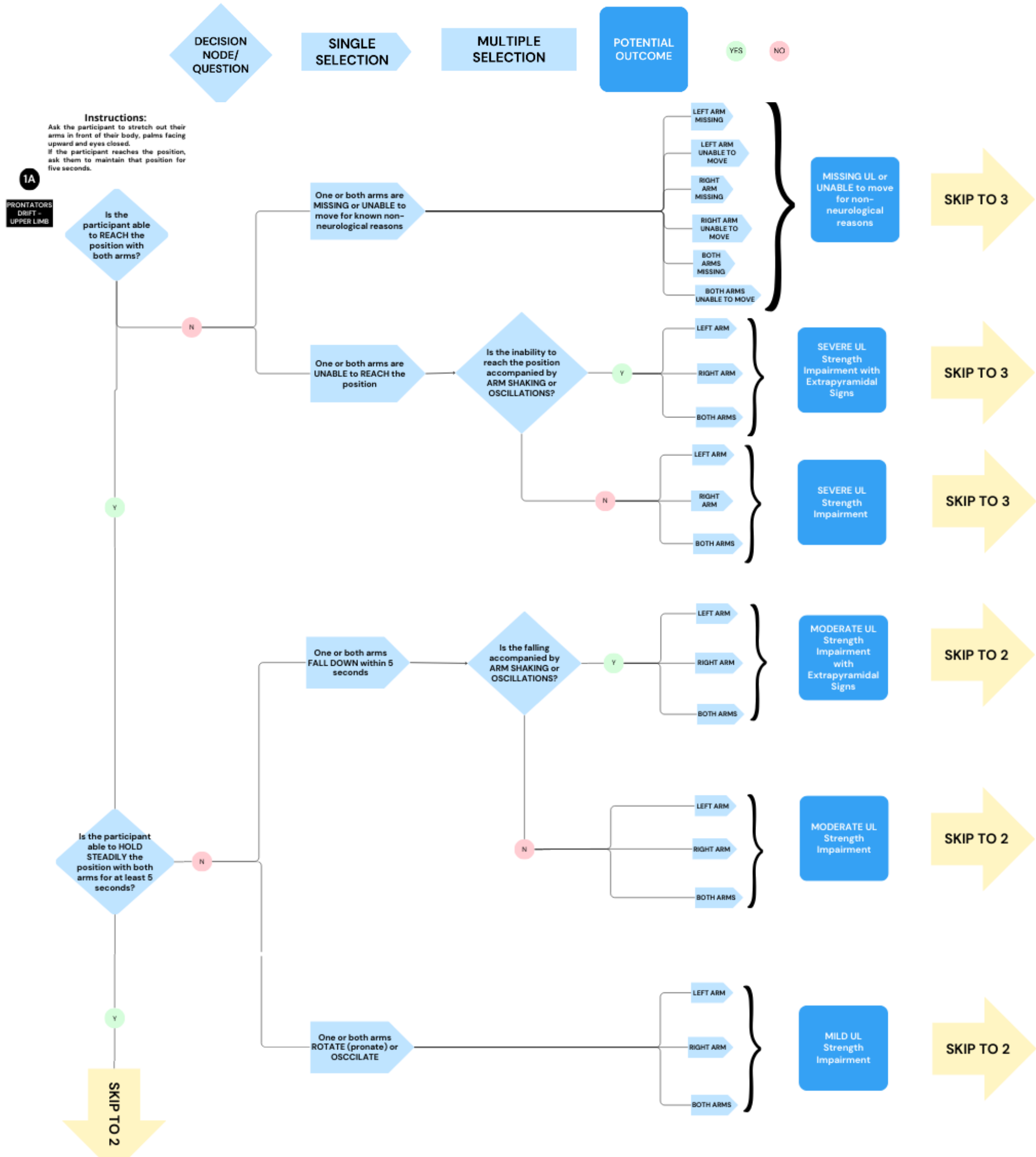

Figure 1. Representation of the step-by-step flowchart in each section. Section 1A – Pronator's Drift of the Upper Limbs. The caption regarding the shapes can be found above. Section 5 is a digital adaptation of a cognitive assessment based on the Mini-Mental State Examination.

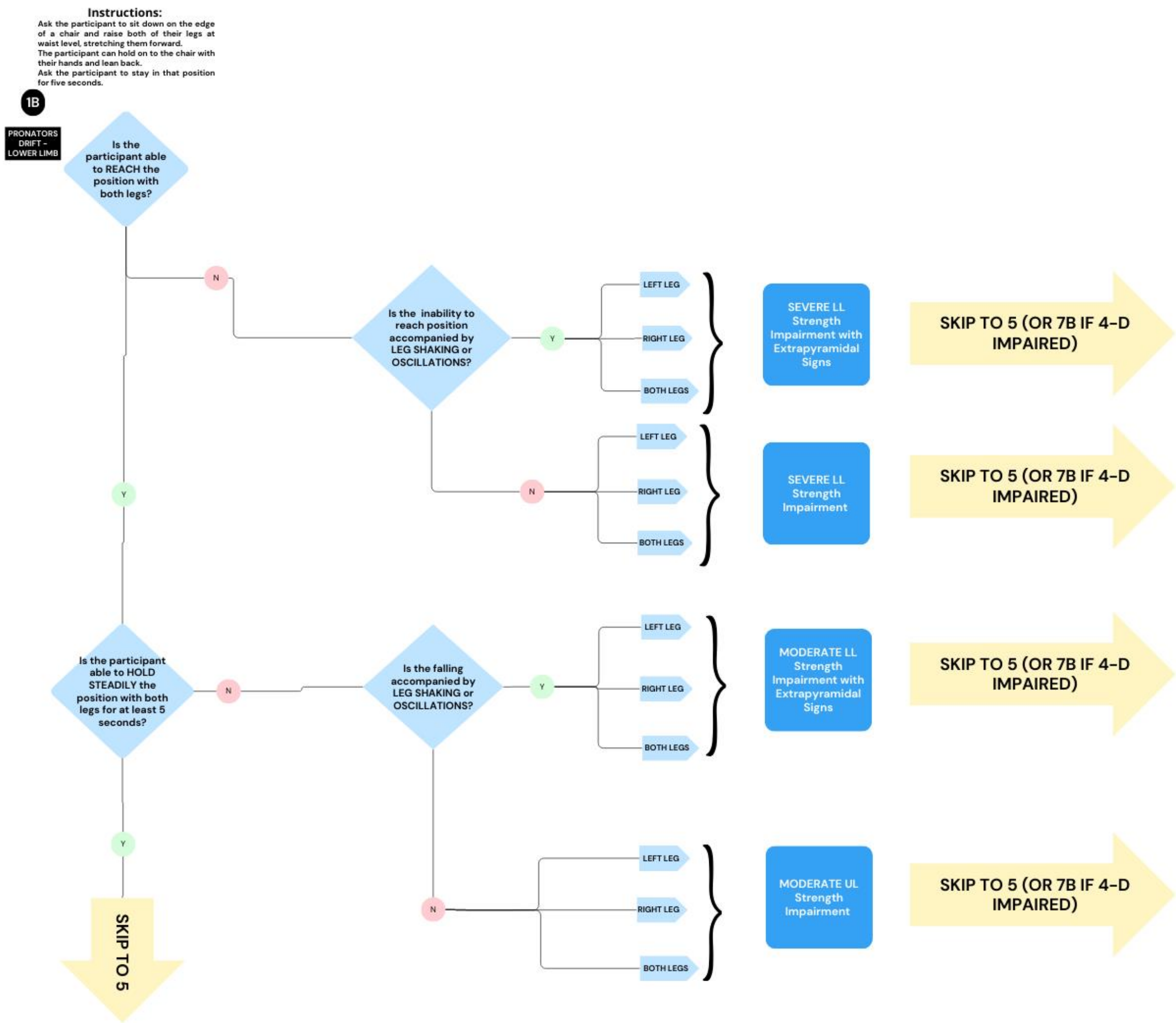

Figure 2. Section 1B of the NeuroEpiTool - Pronator's Drift of the Lower Limbs.

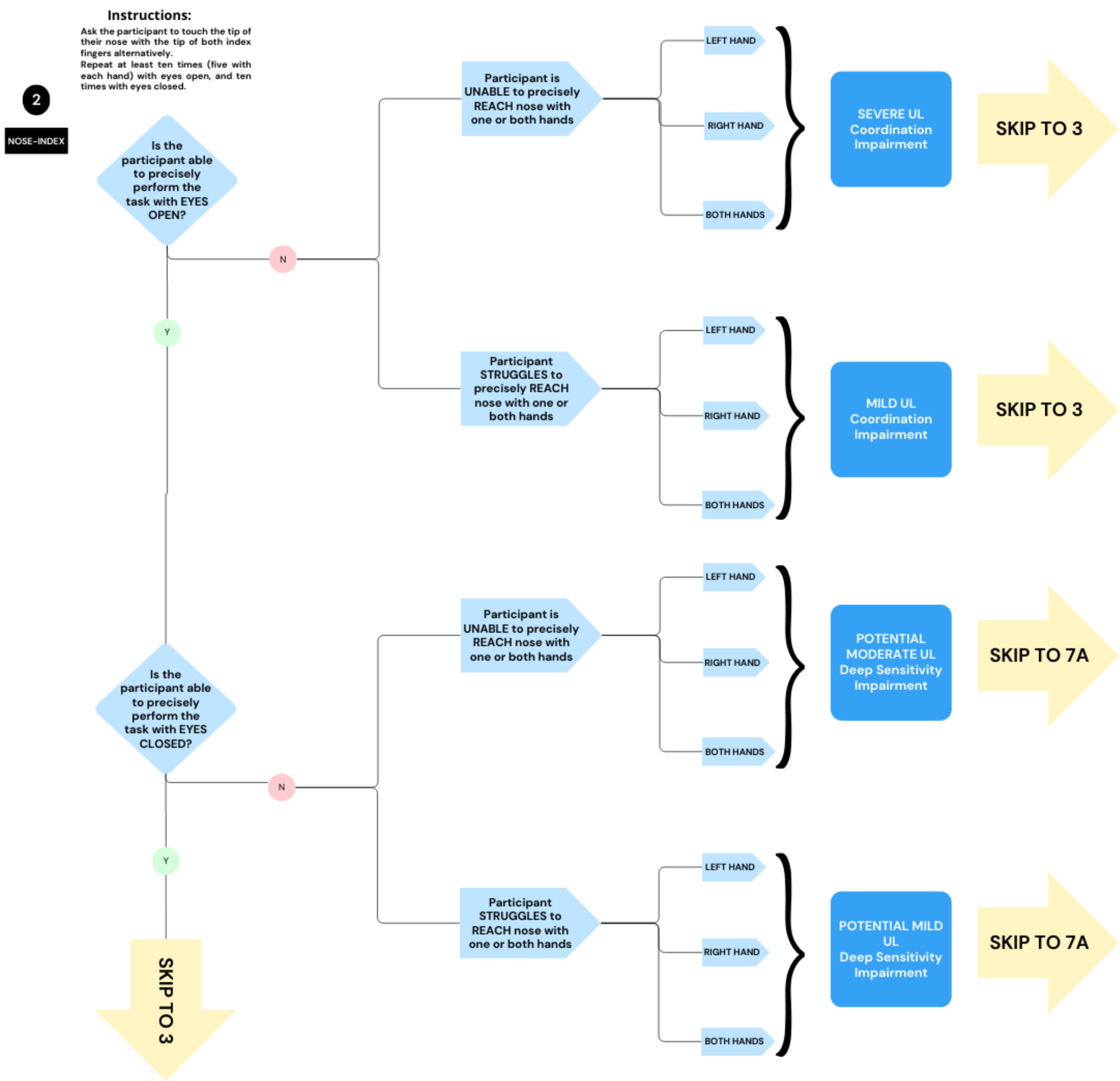

Figure 3. Section 2 of the NeuroEpiTool - Nose-Index.

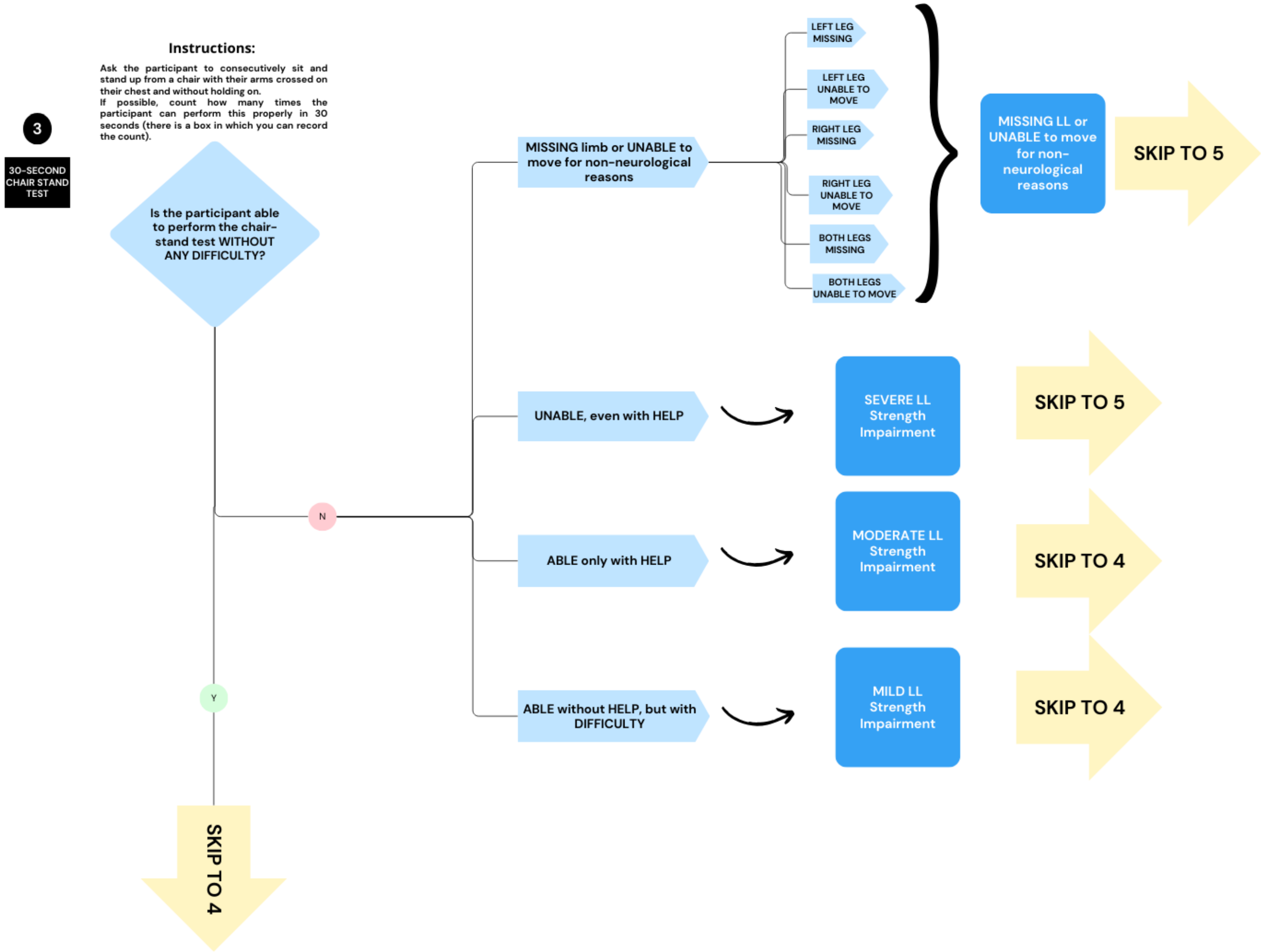

Figure 4. Section 3 of the NeuroEpiTool - 30-second chair stand test.

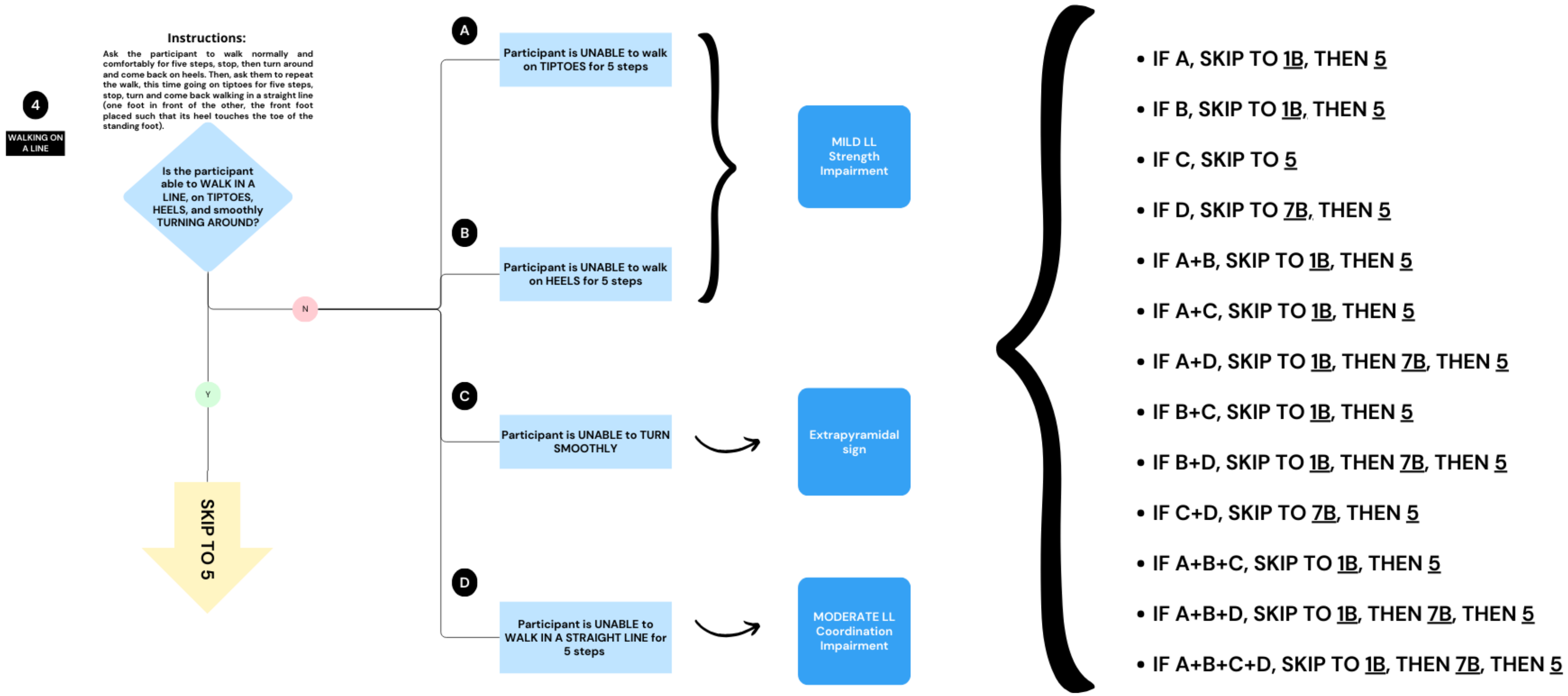

Figure 5. Section 4 of the NeuroEpiTool - Walking on a Line.

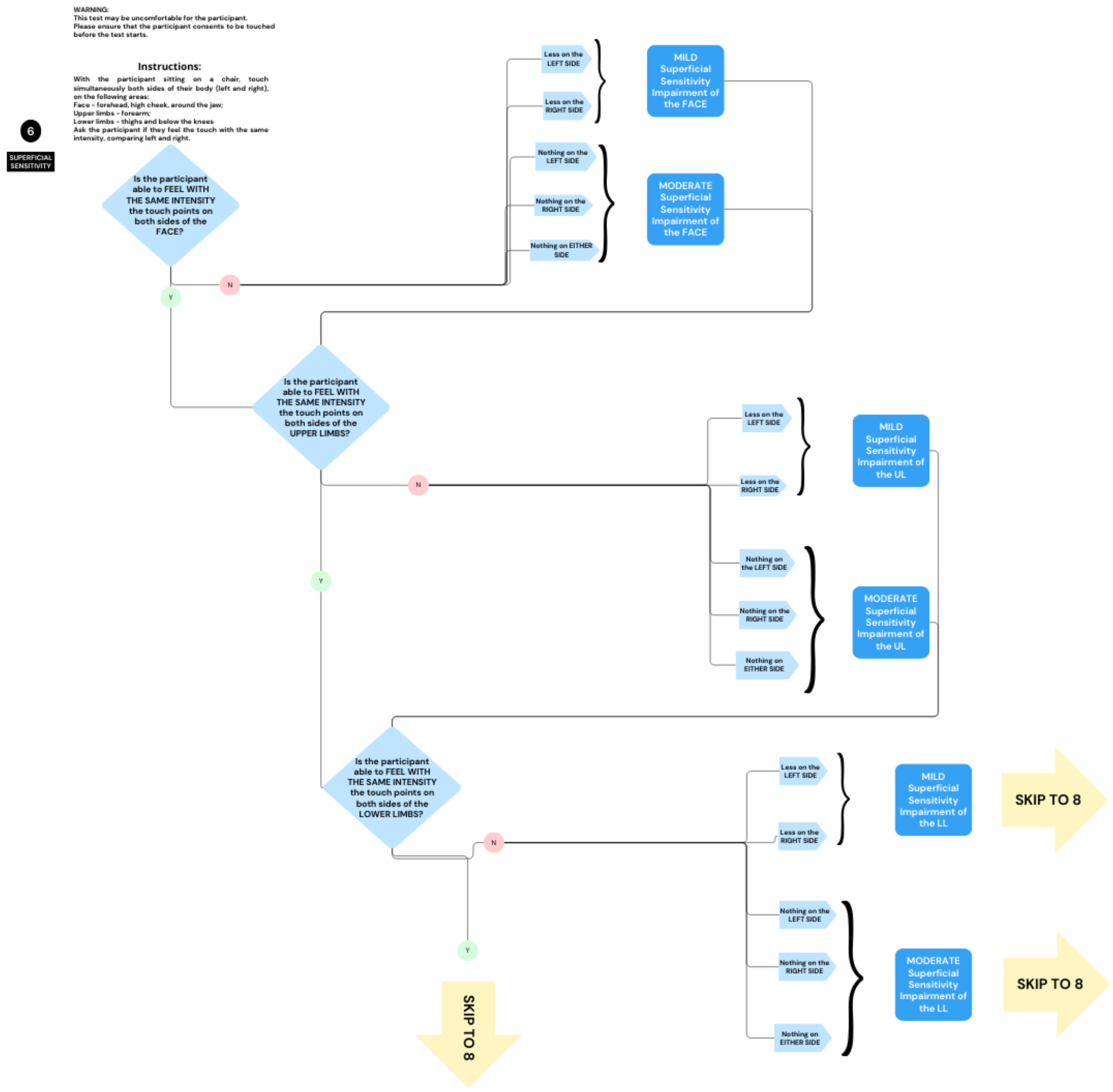

Figure 6. Section 6 of the NeuroEpiTool - Superficial Sensitivity.

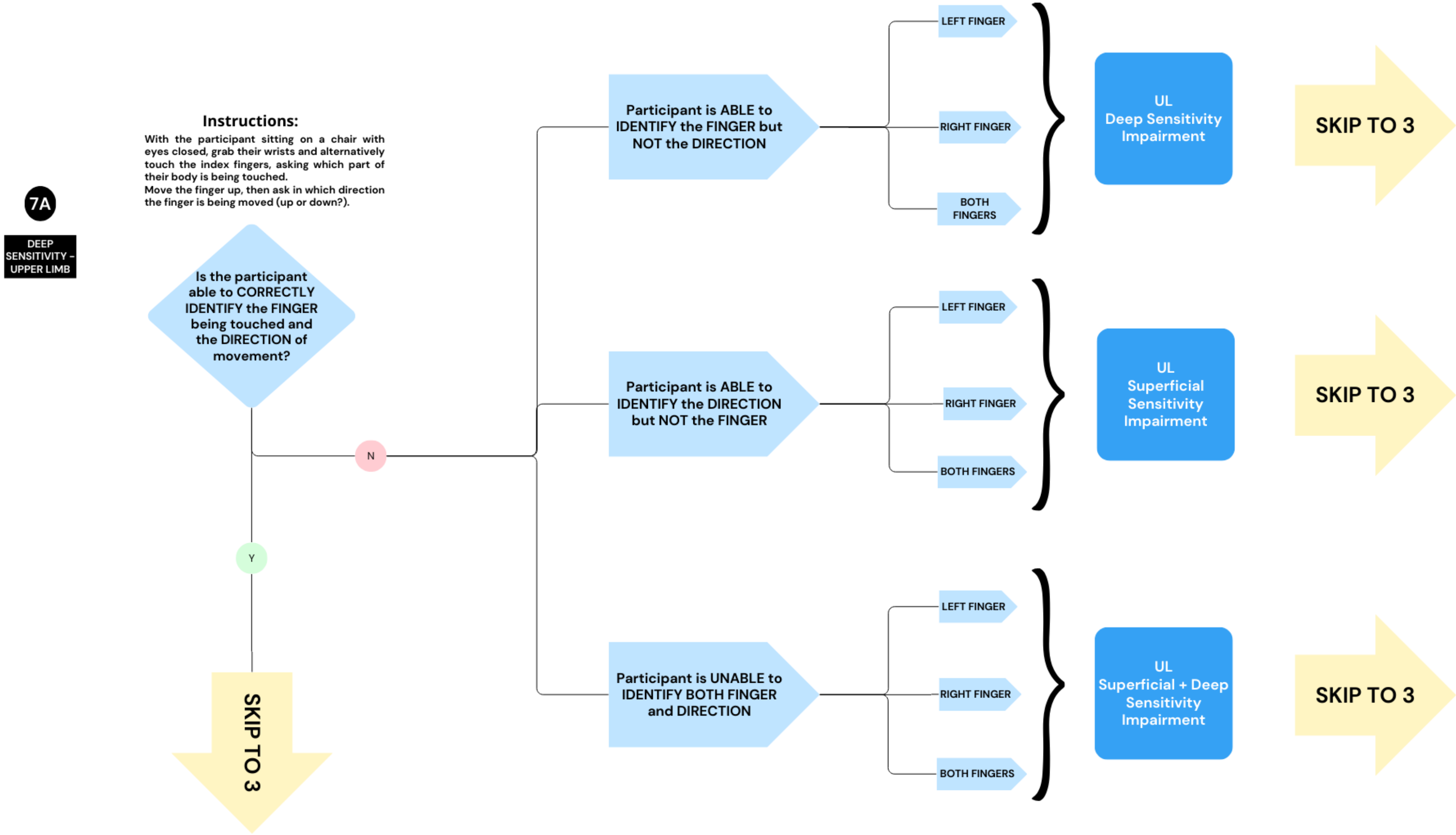

Figure 7. Section 7A of the NeuroEpiTool - Deep Sensitivity of the Upper Limb.

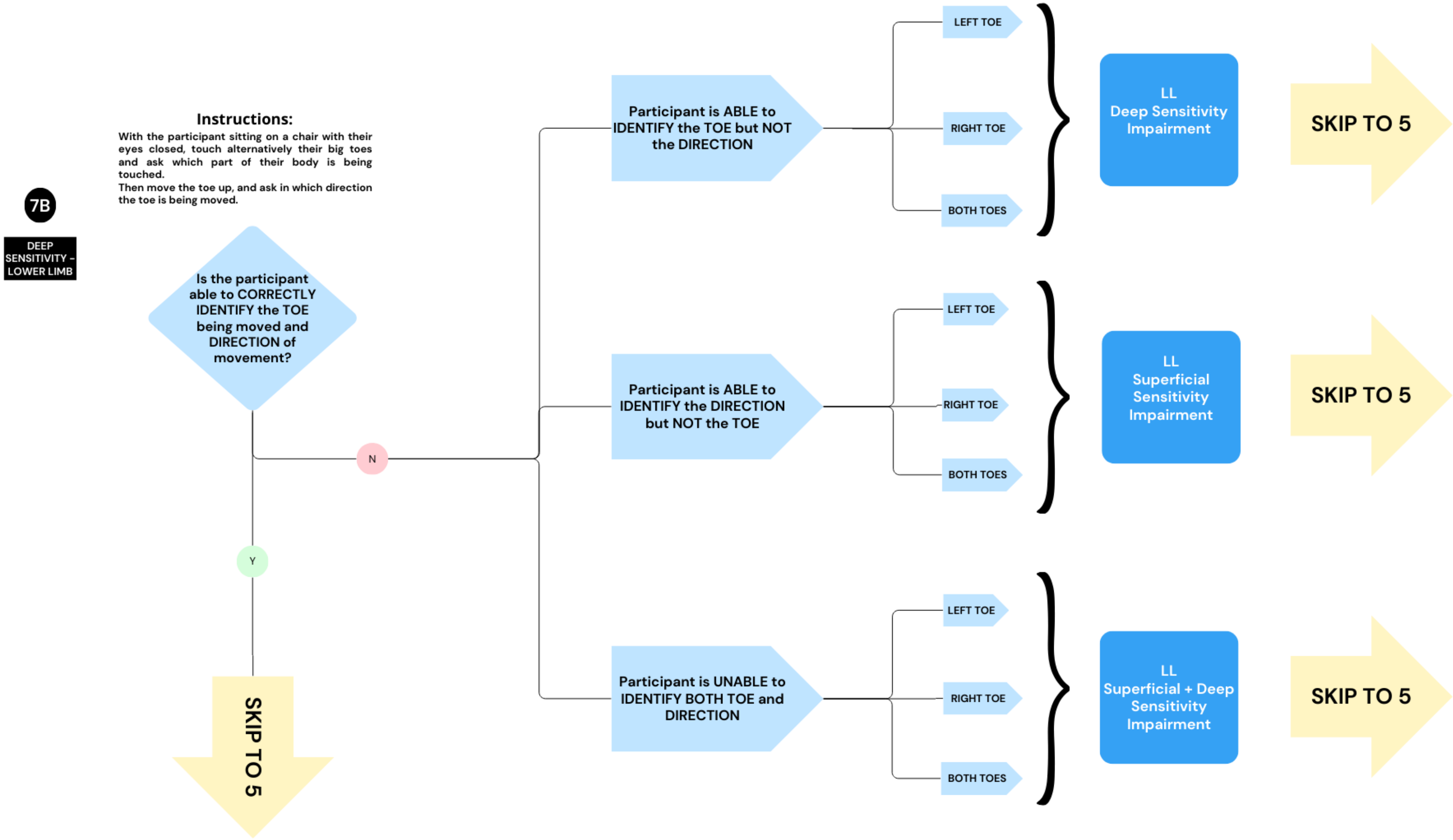

Figure 8. Section 7B of the NeuroEpiTool - Deep Sensitivity of the Lower Limbs.

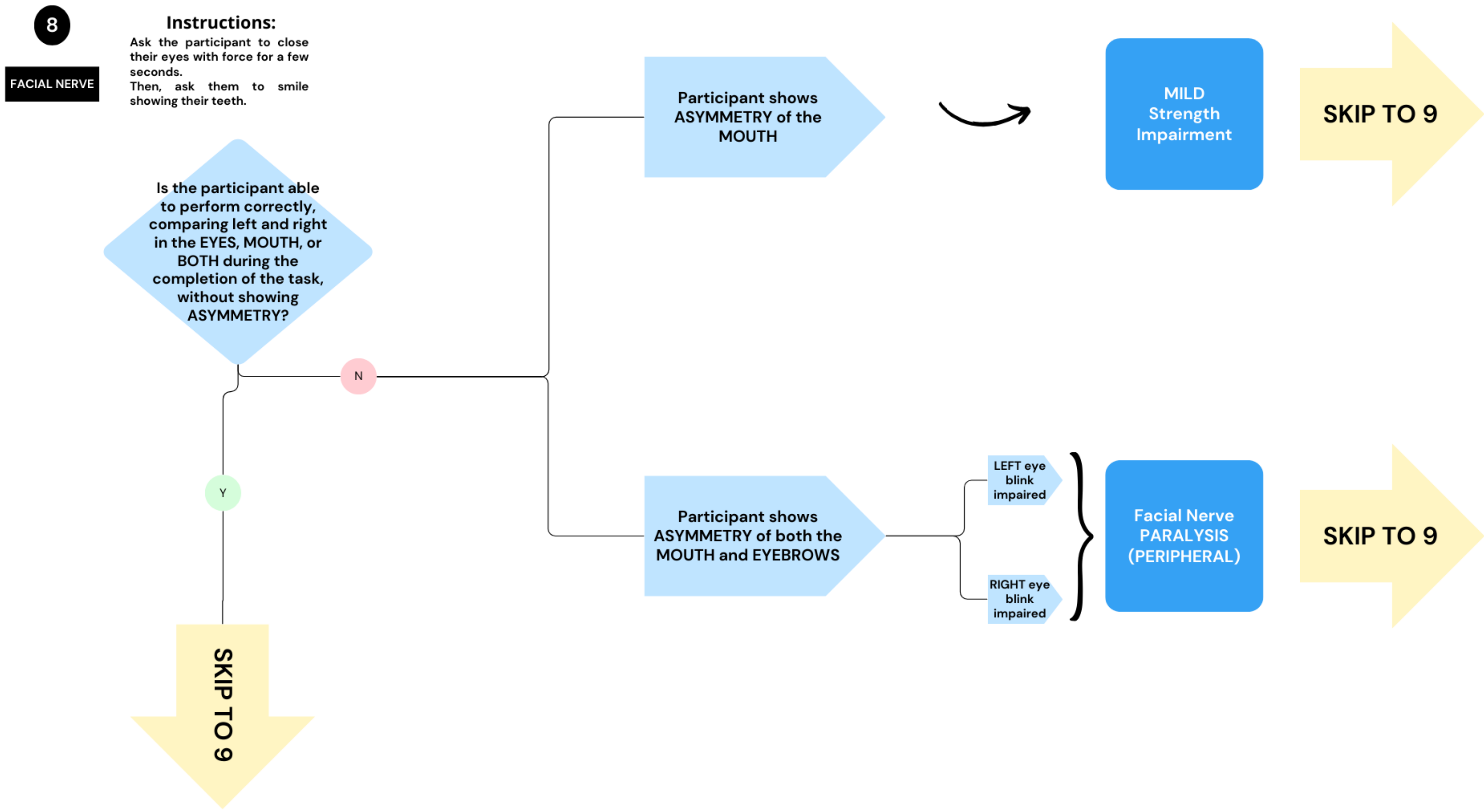

Figure 9. Section 8 of the NeuroEpiTool - Facial Nerve.

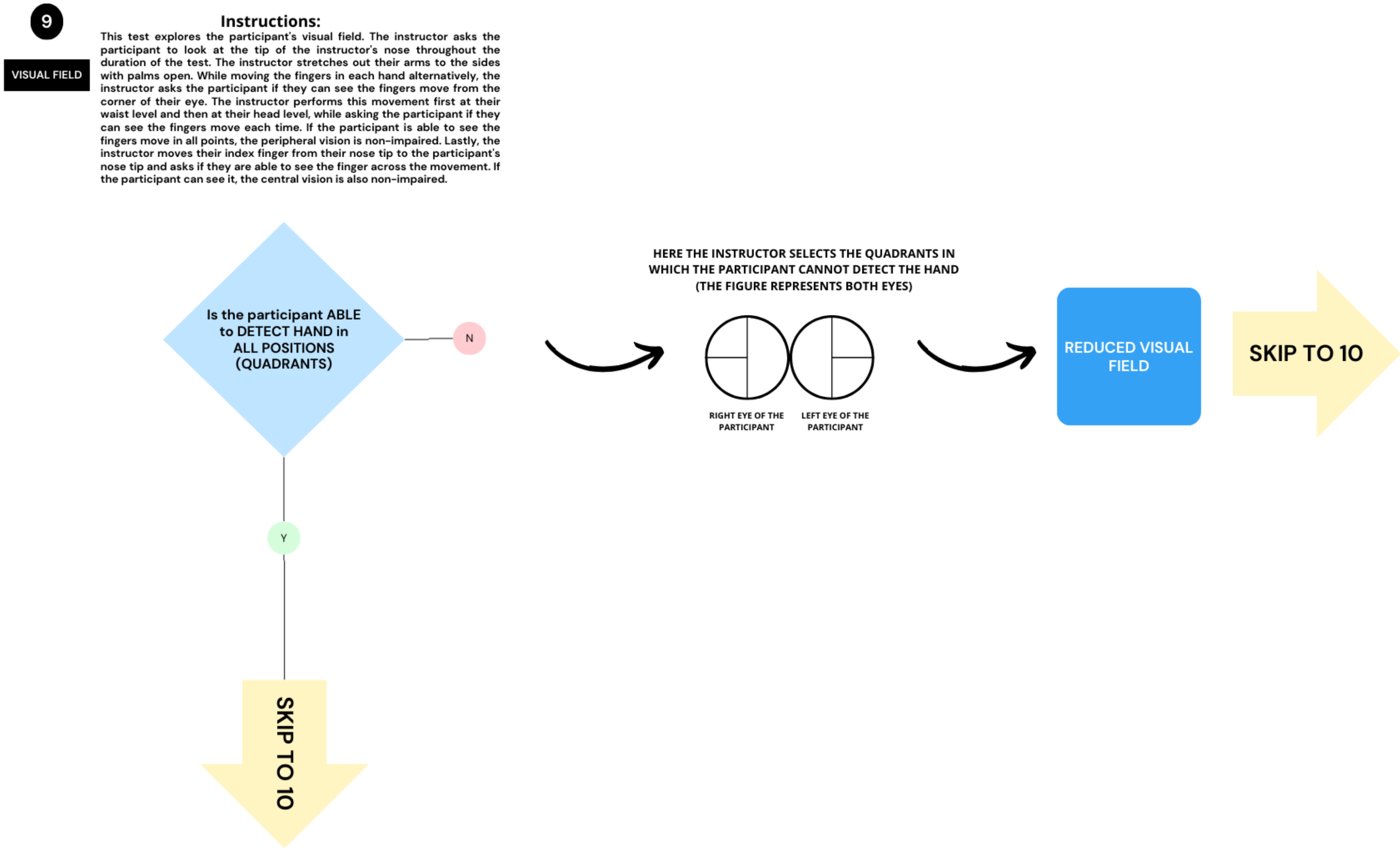

Figure 10. Section 9 of the NeuroEpiTool - Visual Field.

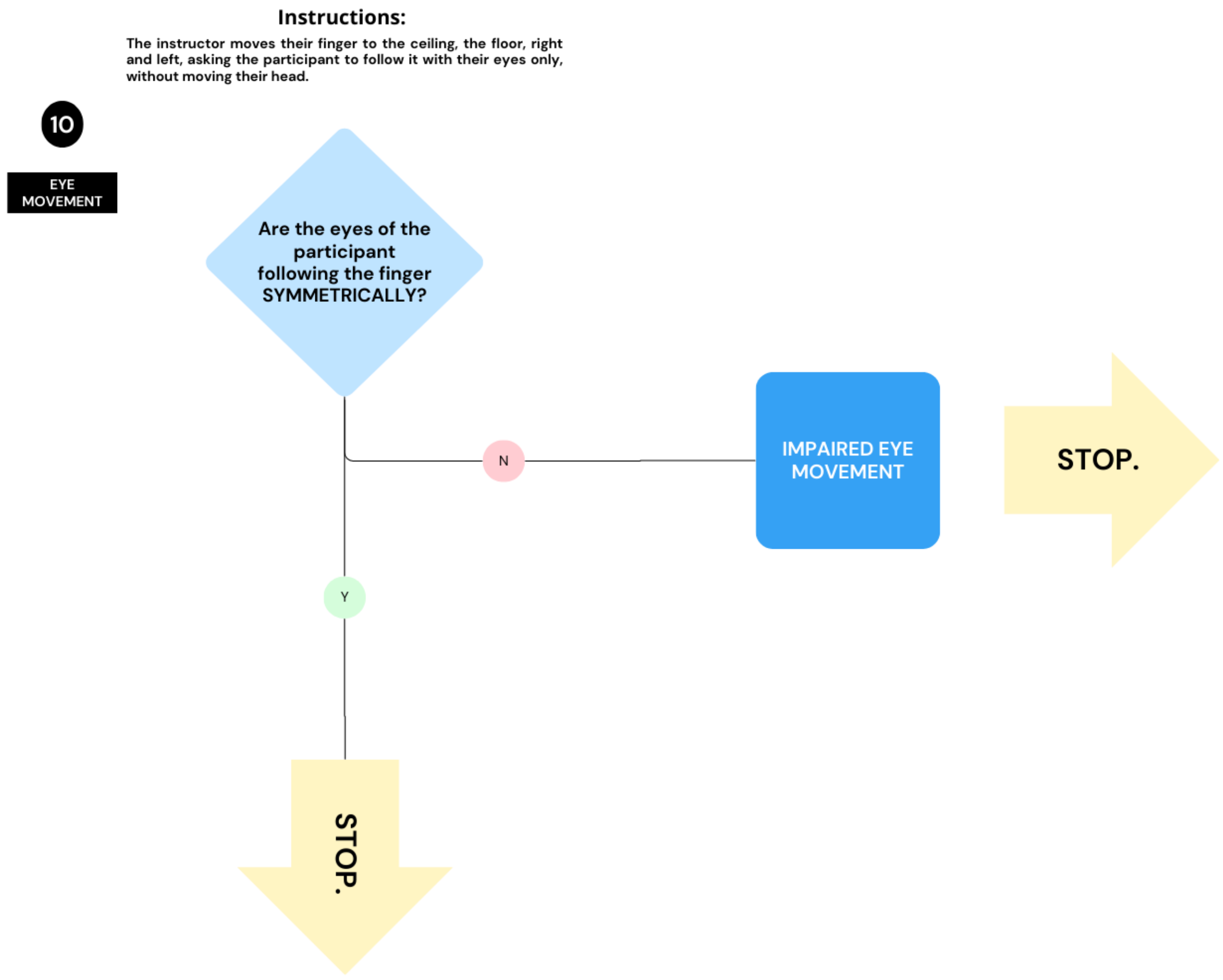

Figure 11. Section 10 of the NeuroEpiTool - Eye Movement.
